# Greener Synthesis of Eco-Friendly Biodegradable Mesoporous Bioactive Glasses with and Without Thermal Treatment and Its Effects on Drug Delivery and In Vitro Bioactivity

**DOI:** 10.3390/ijms26136524

**Published:** 2025-07-07

**Authors:** Dana Almasri, Yaser Dahman

**Affiliations:** 1 Department of Electrical, Computer & Biomedical Engineering, Toronto Metropolitan University, Toronto, ON M5B 2K3, Canada; dmasri1@torontomu.ca; 2 Department of Chemical Engineering, Toronto Metropolitan University, Toronto, ON M5B 2K3, Canada

**Keywords:** bioactive glass, mesoporous, salt template, thermal treatment, synthesis, drug loading, drug release kinetics, kinetics models

## Abstract

This study investigates the use of a salt template to synthesize mesoporous bioactive glass (MBG). Different salts were used as hard templates to create pores in the glass structure to investigate the possibility of using acid-soluble salt templates and to understand the properties of glass synthesized without thermal treatment. The MBGs were synthesized in a TRIS buffer solution at a pH of 9.5 to allow hydrolysis of the metal oxide precursors. The glass was then washed with mild acid to remove the template. After the samples were washed, some were subjected to thermal treatment, while others were not to investigate the impact of thermal treatment on the structure of the MBG. The successful synthesis of MBG was confirmed by X-ray diffraction, Fourier-transfer infrared spectroscopy, scanning emission scanning microscope, and nitrogen adsorption–desorption analysis. This synthesized MBG had a large surface area, pore volume, pore size, and high drug loading efficiency. MBG synthesized without thermal treatment had slower degradation over the test period, but higher loading efficiency and slower drug release, making it appropriate for applications requiring long-term drug delivery while maintaining its bioactivity.

## 1. Introduction

Ever since Hench pioneered the development of bioactive glass in the 1960s, it has captured significant attention in the field of biomaterials research. Bioactive glass (BG), composed of various metal oxides, can be implanted into the human body, gradually dissolving over time and facilitating new bone formation. Bioactive glass synthesis can be achieved through different methods, such as melt-quench or sol–gel techniques, each of which offers unique properties and reaction pathways [1]. One specific type of BG that has gained prominence is mesoporous bioactive glass (MBG) [1]. MBG is a highly porous BG that possesses exceptional properties such as high pore volume, surface area, controlled degradability, and excellent bioactivity [2]. Through the sol–gel method, MBG can be synthesized by introducing surface-directing agents that result in the formation of well-defined pores [3]. When synthesizing MBG, there are numerous available techniques that offer control over pore size, surface area, and pore volume [4,5]. The appeal of MBG stems from its bioactivity and biodegradability [2]. Upon implantation, MBG can dissolve within the body, forming a bone-like apatite layer that fuses with the surrounding tissue and promotes bone repair [4]. Furthermore, the customizable nature of MBG allows for the development of scaffolds and coatings with optimized properties to facilitate bone regeneration, drug delivery systems, and dental applications [2]. In this paper, a new approach for MBG synthesis is explored, and its potential applications in bone tissue engineering and drug delivery are discussed. The synthesized MBG is characterized, and its properties are analyzed, highlighting its advantages over traditional glasses and its potential as a versatile biomaterial [2]. Furthermore, the versatility and wide range of techniques available for MBG synthesis ensure their suitability for various biomedical applications.

Pores created in the bioactive glass are usually produced by surface-directing agents (SDAs) or chemically synthesized surfactants. These chemical surfactants are nanosized or microsized and can be added to the solution to form pores inside the bioactive glass; these SDA templates usually form micelles in water or ethanol when their concentrations reach critical micelle concentrations (CMCs). Although highly porous, mesoporous materials are commonly synthesized using surfactants, which have some downsides, including toxicity [5], leaving residual components [6], their cost and production, limitation in pore size control, and their environmental impact [7]. Finding surface-directing agents that can create mesopores in MBG and be removed without subjecting the bioactive glass to high temperatures would lead to a new green bioactive glass for diverse applications. Conventional MBG synthesis relies on the use of surfactants to form mesopores, which are typically removed by calcination. Calcination is a process that wastes energy and risks damaging the glass structure through pore collapse. The proposed approach offers a more sustainable alternative by reducing energy use and avoiding high temperatures, enabling the incorporation of heat-sensitive drugs and scalable production.

Salt templates have been explored as a replacement for commonly used SDAs as they have several advantages including easy removal, tunability of size, thermal stability, and versatility. The salt template has been successfully used in the synthesis of carbon-based materials, metals, and metal oxides [8]. This study demonstrates the high potential of using a salt template to replace currently employed SDAs, which require removal via high temperatures or harsh ethanol treatment. Salt templates can be removed using mild acid, which can dissolve the salt, leaving the structure of the porous materials mostly unaltered. To investigate the impact of different salts, this study was designed to examine the ability to use water-insoluble salts like Mg(OH)_2_, as well as salts with limited solubility in ethanol like NaCl.

Another aspect of bioactive glass design and synthesis is understanding the impact of high thermal treatment on the properties of the glass. Calcination is often an important step in the synthesis of bioactive glass as it leads to the removal of unwanted or residual remnants of the hydrolysis and condensation reactions [9,10,11]. Calcination involves heating the products of the sol–gel synthesis gradually to 600 °C and keeping it there for around 1 h to remove SDA templates. The need for calcination or thermal treatment has not been investigated before. Although multiple studies look at the calcination rate and thermal treatment impact on structure and properties, none of them discuss the impact of calcination on bioactivity. Some studies have investigated the synthesis of MBG without calcination but have not investigated it in relation to MBG that has been calcined [12]. This present study introduces a novel approach for developing MBGs by utilizing water-insoluble salts as hard templates, eliminating the need to organize solvents or complex removal processes. Unlike conventional methods, this work systematically compares the effect of thermal treatment versus non-thermal routes on bioactivity, drug loading, and release kinetics, which will provide new insights into MBG that is optimized for biomedical applications.

## 2. Results and Discussion

**Figure 1:**
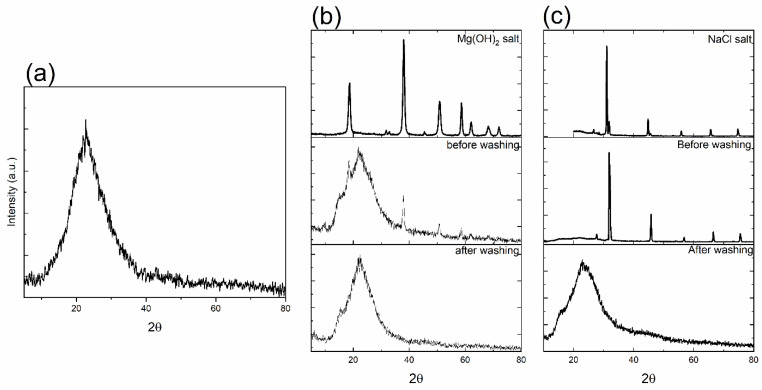
The X-ray diffraction spectra of as-synthesized MBG where (**a**) shows the spectra for MBG-C; (**b**) shows the spectra for Mg(OH)_2_ salt, MBG-M before washing, and MBG after washing; and (**c**) shows the spectra of NaCl salt, MBG-N before washing, and MBG after washing.

Figure 1 shows the X-ray diffraction patterns of all synthesized MBG samples synthesized using salt templates. Figure 1a shows the calcined sample that underwent thermal treatment to remove the salt template, Mg(OH)_2_. Figure 1b,c show non-calcined samples synthesized using two different salt templates: Mg(OH)_2_ (MBG-M) and NaCl (MBG-N). For these samples, the templates were removed through mild acid addition, which dissolved the templates while preserving the MBG structure. All spectra in Figure 1 showed the asymmetrical diffuse halo around the diffraction angle of 20°, indicating the amorphous structure of the synthesized MBG [13]. Figure 1b,c show crystalline peaks that match the peaks of the salt spectra, which means that the MBG was successfully synthesized using the salt template. After it was washed with mild acid, the XRD spectra showed that the salt was removed from MBG, and the only thing left was the synthesized glass. Notably, the broad peak for MBG-N appears smaller before washing which occurs because the high-intensity NaCl peaks overshadow it, making the broad peak seem smaller compared to pre-washed spectra of MBG-M.

When comparing the MBG that underwent calcination and the one that was washed using acid, it is evident that the glasses show a similar amorphous pattern that is characteristic of bioactive glass. There are no significant differences in the XRD spectra between the two of them. There is a broad dispersive amorphous peak that starts at 10° and ends around 38°. This dispersive peak is also observed in other studies as well [14,15].

**Figure 2:**
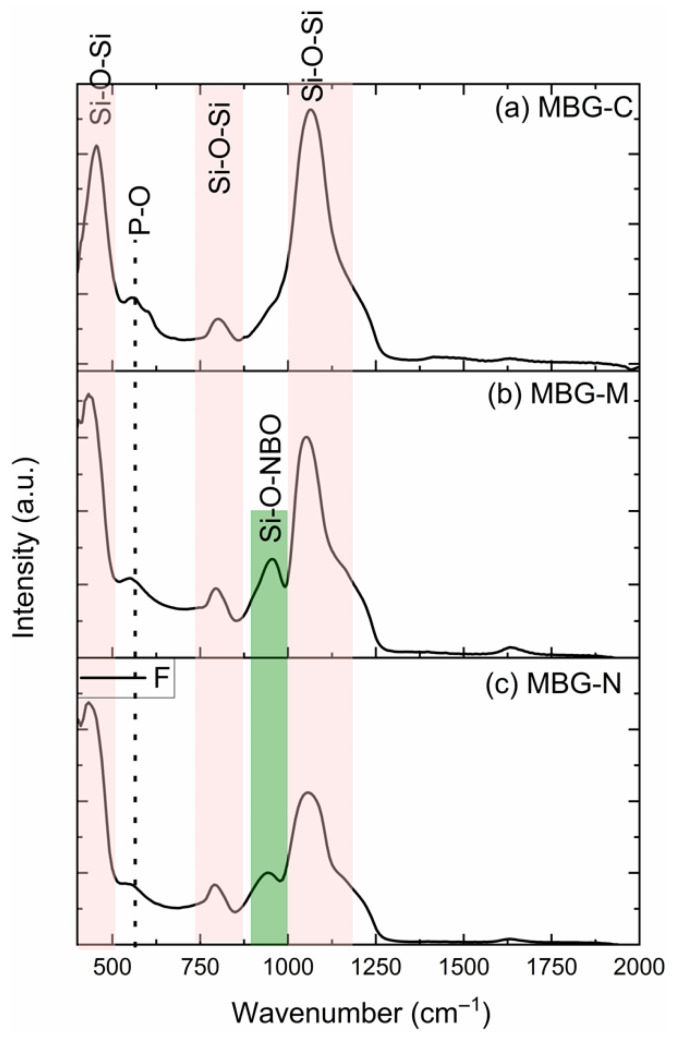
The FTIR spectra of (**a**) MBG-C, (**b**) MBG-M, and (**c**) MBG-N. The spectra show the silicon bonded to bridging oxygens (Si-O-Si) and silicon bonded to non-bridging (Si-O-NBO) as well as the P-O bonds as observed in the synthesized glass.

FTIR was used to analyze the chemical bonding and phase composition of all MBG samples. Figure 2 shows the FTIR spectra collected for MBG-C (a), MBG-M (b), and MBG-N (c), which shows similar patterns for the three MBG samples. The peaks at 450, 760, and 1070 cm^−1^ for the MBG treated by calcination resemble the spectra of previously synthesized bioactive glass as shown in [16], indicating the successful synthesis of bioactive glass using a salt template. The peak at 450 cm^−1^ represents the bending vibration of Si-O-Si, while the one at 1070 cm^−1^ represents the asymmetric stretching vibration of the Si-O-Si. The band at 760 cm^−1^ represents the symmetric stretching of Si-O-Si. There is a weak band at 550 cm^−1^, which is usually attributed to the stretching mode of P-O [17]. However, there is a peak on MBG-M and MBG-N observed around 960 cm^−1^, which is not usually observed in calcined bioactive glass before immersion in SBF or PBS. This peak represents the bond between Si and the non-bridging oxygen, as discussed in [18]. The peak at around 960 cm^−1^ represents the Si-O non-bridging oxygen stretching mode [17], which is only observed here when the samples did not undergo thermal treatment.

The structure of bioactive glass differs from pure silica due to the incorporation of modifying metal oxides. These oxides introduce non-bridging oxygens (NBOs) into the network, which happens when oxygen atoms are not bonded to two silicon atoms [19]. While NBOs typically enhance bioactivity by weakening the silica network structure and dissolution rates [20,21]. In this study, it was observed that the non-calcined samples demonstrated an exception to this trend. Despite containing NBOs, these samples exhibited slower degradation; this is likely due to their unprocessed structural state. This suggests that calcination serves multiple purposes beyond merely removing impurities and stabilizing the structure; it also plays a crucial role in determining bioactivity [3,22].

**Figure 3:**
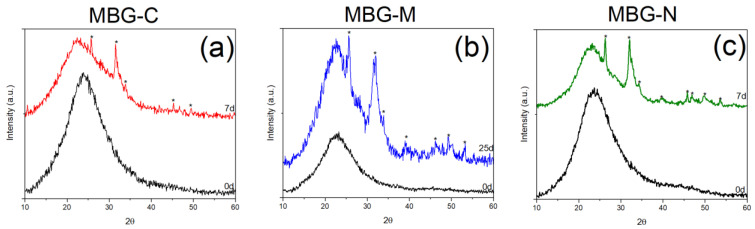
XRD pattern of synthesized MBG before and after immersion in SBF. (**a**) shows the peaks for MBG-C, (**b**) shows the peaks for MBG-M and (**c**) shows the peaks for MBG-N The figure shows peaks highlighted with * that indicate the development of HA on the surface.

Bioactivity of all MBG samples was examined using XRD and FTIR by showing the development of hydroxyapatite (HA) on the surface of the MBG. Figure 3 shows the XRD spectra collected for the different samples. The XRD patterns for MBG-C, shown in Figure 3a, show crystalline peaks characteristic of HA formation. These peaks agree with the standard HA pattern from the JCPDS PDF no. 01-086-0740 [23]. The observed peaks that appeared at 2θ of 25.7°, 31.5°, 39.9°, 45.3°, 49.3°, and 53.1° are indicative of the development of HA on the surface of MBG after SBF immersion. These peaks correspond to specific crystallographic planes in the HA structure: 25.7° (002), 31.5° (211), 39.9° (310), 45.3° (222), and 53.12° (213). The presence of these well-defined peaks, particularly the prominent (002) and (211) reflections, confirms the formation of a crystalline HA layer, which is a key indicator of the bioactive nature of MBG [23].

Figure 3b shows the bioactivity of MBG-M which exhibited crystalline peaks of HA on its surface, similar to the calcined sample. These peaks were observed at 25.6°, 31.5°, 32°, 33.9°, and 39°, representing the (h, k, l) diffraction planes (002), (211), (300), and (310), respectively. Additionally, peaks appearing at θ, 45.3°, 46.2°, 49.3°, 53.3°, represent the (203), (222), (213), and (004), (422), respectively [24]. For this MBG, the development of the crystalline phase took a longer period of time.

Figure 3c shows the XRD of the sample synthesized using NaCl which shows crystalline HA peaks just seven days after immersion in SBF. These peaks are observed at 2θ of 26.3°, 32.1°, 39.6°, 45.8°, 46.9°, 49.8°, and 53.6° which correspond to the crystallographic planes in HA structure as mentioned before [24]. The XRD analysis reveals more prominent peaks for MBG-N, attributed to its rapid bioactive response and enhanced dissolution kinetics in SBF. This accelerated formation of HA on the glass surface, which is a well-established marker of bioactivity confirming the material’s suitability for bone regeneration applications [25]. These observations align with previous studies on sol–gel derived bioactive glasses, where HA crystallization typically initiates within 2 days of SBF immersion, with peak intensity and resolution progressively increasing with longer soaking durations [17,26].

The results here are consistent with HA developing on the surface of bioactive glass as observed by the diffraction peaks at 25.87° (002), 31.77° (211), 32.19° (300), 32.9° (310), and 39.81° (222); 2θ values are characteristic of crystalline HA, matching standard reference patterns from the International Centre for Diffraction Data (ICDF) 09-0432) [23]. Additional peaks observed at 45.3° (203), 46.5° (213), 49.4° (004), and 53.2° (422) further verify HA formation [27].

**Figure 4:**
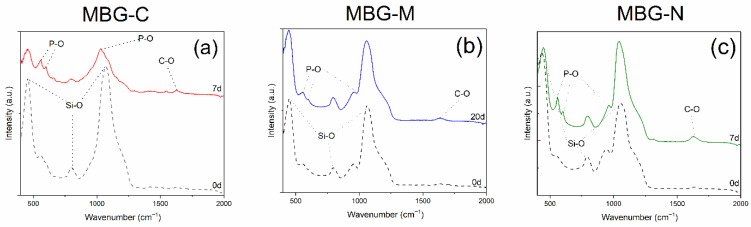
FTIR results of the MBG synthesized with salt template and with or without calcination after immersion in SBF. (**a**) shows MBG-C after immersion in SBF for 7 days, (**b**) shows MBG-M after immersion in SBF for 20 days, and (**c**) shows MBG-N after immersion for 7 days.

Figure 4a shows the FTIR spectra of the MBG-C; peaks are observed at 451, 559, 600, 797, 961, and 1069 cm^−1^ before immersion in SBF. After immersion in SBF, new peaks are observed including peaks that appeared at 557.23 and 598.23 cm^−1^ which represent the vibrational modes of P-O bonds. Peaks at 1293.38 and 1623.25 cm^−1^ are attributed to C-O bonds. Evidence of glass dissolution is observed through the peak shift from 1069.74 cm^−1^ to 1032.47 cm^−1^. All new peaks confirm the development of HA on the surface of MBG.

Figure 4b shows the bioactivity of MBG-M including peaks before immersion at 447.28, 544.19, 797.65, 946.74, and 1058.56 cm^−1^. These peaks correspond to the bond of Si-O which are categorized into bonds between silicon and bonding oxygen (447.2805, 797.6503 and 1058.5639 cm^−1^) and silicon with non-bonding oxygens (946.7438 cm^−1^). After immersion in SBF, new peaks appear at 551.646, 626.1928, 1148.021, and 1200.2028 cm^−1^. These peaks appear as HA develops on the surface of the bioactive glass. Peaks 551.646 and 626.1928 cm^−1^ represent P-O bonds for HA, while 1148.021 and 1200.2028 cm^−1^ show C-O bonds in HA. This is consistent with results observed in Jones et al. (2003) [28] and Yan et al. (2005) [29].

Figure 4c shows bioactivity of the MBG-NaCl which has peaks at 432.37, 790.19, 946.73, and 1058.56 cm^−1^ before immersion. After immersion in SBF, peaks are observed at 447.23, 559.10, 596.37, 797.65, 961.65, 1043.65, and 1625.11 cm^−1^ which can be attributed to stretching modes of PO_3_^4−^ as mentioned before. Glass dissolution is evidenced by the shift in peaks at 1058.56 to 1043.65 cm^−1^ and 43.37 to 447.23 cm^−1^ and finally 946.73 to 961.65 cm^−1^. These new peaks indicate the development of HA crystals on the surface of the MBG, while the shifts indicate the dissolution of the glass network. This was observed within 7 days which means that this glass would be ideal for slow delivery of antibiotics. It is also important to note that the intensity of the Si-O bonds decreased as the glass network dissolved: the peak at ~450 cm^−1^ decreased in intensity from 0.609 to 0.328, the peak at ~1060 cm^−1^ decreased from 0.708 to 0.324, and the peak at ~950 cm^−1^ decreased from 0.113 to 0.056.

The FTIR results of the three samples are consistent with previous studies, where HA deposition on the surface was proven by the HA crystal peaks as also observed here at 551.646 and 626.1928 cm^−1^ [14]. Furthermore, the presence of Si-O NBO stretching vibrations in uncalcined samples matches the observations reported by Shah et al. (2015) [19], confirming the retention of silicate network modifications prior to thermal processing.

Figure 3 and Figure 4 show the bioactivity of MBG. Bioactivity is defined as the ability of a material to elicit a biological response when it comes in contact with biological or living tissue [22]. In terms of MBGs, bioactivity can be characterized by their ability to form HA on their surface in physiological fluid, which is important for osseointegration and stimulation of osteogenic processes [30]. Results show that the different formulations had HA development at different times, where MBG-M showed the slowest bioactivity out of the three, as was expected due to the lack of calcination. It was expected for MBG-N to have a similar bioactivity, but that was not the case as MBG-N showed signs of HA development after 7 days, which means that when using salts as surface-directing agents, the salt itself may impact the structure of the MBG. Consistent with previous studies, MBG-C showed HA development after a few hours of immersion in SBF which means that thermal treatment is an important factor in the synthesis of MBGs if they are designed for drug delivery that needs quick release and fast degradation to re-integrate with the bones, while MBG-M would be excellent for applications that require slower release and slower degradation. For example, in cases of biofilm, which is a highly antibiotic-resistant bacterial infection that is very prevalent after orthopedic surgeries, the need for biomaterials that can have high drug loading and slow drug release is crucial [31].

Biocompatibility of MBG has been established in previous studies. In vitro tests conducted by Chen et al. (2018) [32] demonstrated that 58S BG is biocompatible by testing the cell viability and cultivating MC3T3 osteoblasts cells with BG and observing their viability. The study found that cells grow well after culturing with BG. Another study was conducted by Anand et al. (2019) [23], in which they tested biocompatibility of MBG in vivo and found that all samples they tested led to the formation of new bones and MBG led to new bone formation at 68.2% after 90 days. Another comprehensive review conducted by Salètes et al. (2021) [33] examined the biocompatibility of MBGs based on in vitro studies. The review examined different approaches to assess biocompatibility and found that MBG shows favorable biocompatibility, cytocompatibility, and osteoconductive properties and a lack of toxicity [16].

**Figure 5:**
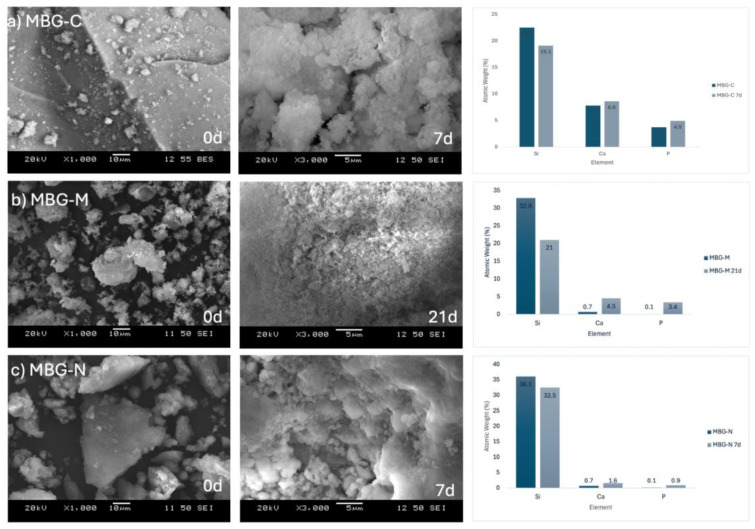
SEM images of (**a**) MBG-C at day 0 and day 7 after immersion in SBF, (**b**) MBG-M at day 0 and day 21, and (**c**) MBG-N at day 0 and day 7. The figure also shows the EDX results for the different MBG at day 0 as well as day 7 for MBG-C and MBG-M or 21 for MBG-M.

SEM analysis shows the bioactivity progression of various samples at different times following immersion in SBF. This technique is widely used to assess the bone-bonding potential of biomaterials [34]. Figure 5 shows the different MBG samples before and after immersion in SBF. Figure 5a shows the development of HA on the calcined sample’s surface after a week in SBF. Initially, the particles appear solid without visible degradation. However, the subsequent image shows evidence of HA formation, a crucial indicator of bioactivity [20]. This observation is corroborated by EDX data, which reports atomic percentages of Si, Ca, and P as 19.1, 8.6, and 4.9, respectively, after 7 days in SBF, yielding a Ca/P ratio of 1.76. This ratio approaches that of stoichiometric HA (1.67), suggesting the formation of a calcium phosphate layer [35]. In comparison, pre-immersion data showed atomic percentages of 22.5, 7.8, and 3.7 for Si, Ca, and P, respectively.

Figure 5b shows MBG-M before and after extended SBF immersion, as this formulation exhibited slower degradation compared to the others. This slower rate aligns with studies demonstrating that lack of thermal treatment can influence the degradation kinetics of bioactive glasses [36]. The SEM images show gradual HA development on the bioactive glass surface, further evidenced by shifts in metal ion atomic percentages. Initially, Si, Ca, and P were at 32.8, 0.7, and 0.1, respectively. After 21 days of SBF immersion, these values changed to 21.0, 4.5, and 3.4, indicating ongoing ion exchange and HA formation processes [21]. EDX data also show that there was no Mg in the structure of the synthesized MBG, confirming that while the salt was used as a template it did not integrate into the structure.

Figure 5c shows the MBG-N sample, which shows the atomic percentages at day 0 were 36.1 for Si, 0.7 for Ca, and 0.1 for P. Post-immersion, these values shifted to 32.5, 1.6, and 0.9, respectively. These compositional changes across all samples are consistent with the characteristic bioactive behavior of MBGs, involving ion exchange with the surrounding medium and subsequent formation of a bioactive surface layer [14]. These findings align with previous studies demonstrating that increased Ca and P concentrations on the bioactive glass surface indicate HA formation. Adams et al. observed HA development on bioactive glass surfaces, evidenced by rising phosphate and calcium concentrations and a decline in silicon after several days of immersion in SBF [37].

The degradation of the MBG samples was first evaluated by monitoring HA formation on their surfaces using FTIR and XRD. Additionally, degradation was assessed by measuring mass loss before and after 21 days of immersion in SBF. MBG-C had the most significant mass change, losing around 60% of its starting mass after immersion, which correlates with the higher ion release observed in previous analyses, indicating a more rapid biodegradation process and higher bioactivity as observed previously. MBG-N had around 52% mass loss, and MBG-M had around 50% mass loss. These results are consistent with the ICP results where the measured ion concentrations in SBF directly reflect each material’s biodegradation rate. Controlled mass loss in bioactive glass is important for bone regeneration to promote sufficient ion release while maintaining structural integrity over time [38]. This balance is essential for ensuring that the material can support new bone formation while gradually degrading to allow for natural tissue integration [39].

**Figure 6:**
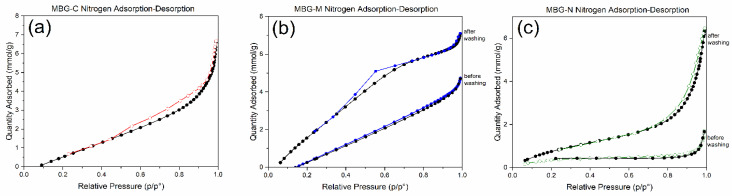
Nitrogen adsorption–desorption of (**a**) MBG-C, (**b**) MBG-M before and after washing, and (**c**) MBG-N before and after washing.

Figure 6a shows an MBG calcined sample which has a type IV isotherm with H4 hysteresis which represents a slit-shaped pore inside the bioactive glass; this type of pore is typical of micro-mesopores materials. Figure 6b shows a hysteresis loop with a H1 shape indicative of well-defined cylindrical pore channels of uniform spheres. Compared to the MBG before and after washing, this shows that before washing the pores are closed with the salt template particles and blocked pores, while after washing the material shows a type IV isotherm with a H1 hysteresis loop indicating successful removal of the salt template. Figure 6c shows the MBG made using NaCl which also shows a type IV isotherm with a H4 hysteresis loop [40], which also indicates the presence of micropores and mesopores with a slit-like opening in the materials or hierarchical structure in the MBG. Compared to the sample before washing it is observed that the pores were closed with the salt template but were opened after washing, indicating a successful synthesis.

Comparing the different MBGs, the figure shows that all synthesized MBGs had a type IV isotherm, which is usually observed for mesoporous materials. Closure at P/Po ~0.4 indicates the presence of small mesopores, which was observed in all formulations, while hysteresis shows capillary condensation in mesopores and macroporous materials. Different hysteresis was observed for different formulations, with MBG-M showing a type H1 hysteresis loop usually observed for materials with more uniform mesopores, while the calcined sample and MBG-N show H4 hysteresis loops, which are more typical of micro-mesoporous materials [40]. These findings are consistent with previous studies on MBGs. Bai et al. [41] reported similar type IV isotherms, confirming the mesoporous structure of MBGs. Additionally, Almasri et al. [25] demonstrated that drug-loaded bioactive glass maintained comparable isotherm profiles to pre-washed samples, while subsequent drug removal resulted in more open isotherms, clearly revealing the material’s porous architecture once the template is removed.

Table 1 shows the surface area, pore volume, and pore sizes of MBG-C, MBG-M, and MBG-N. The surface area of MBG-C was measured to be 201.41 m^2^/g while the pore volume and size were measured to be 0.33 cm^3^/g and 6.56 nm. These measurements are consistent with previous studies [42] and [29], indicating the successful synthesis of MBG using a salt template. MBG-M shows a surface area of 74.37 m^2^/g before washing the salt template which increases to 426.91 m^2^/g after washing; this increase is indicative of the successful washing of the salt templates clearing the pores and increasing the surface area as a result. The pore volume increases from 0.14 to 0.36 cm^3^/g after washing which also shows the clearing of the pores after washing them, making the measurements more consistent with previous studies [29]. Pore size also increased for MBG-M, going from 3.75 nm to 7.41 nm, suggesting the removal of pore blocking salt template. MBG-N shows similar results where the removal of the salt template led to the increase in surface area from 46.59 m^2^/g to 174.24 m^2^/g, the increase in pore volume from 0.14 to 0.26 cm^3^/g, and finally the slight increase in pore size from 5.75 to 5.96 nm. Interestingly, MBG-N exhibited a less pronounced increase in all properties compared to MBG-M, suggesting that Mg(OH)_2_ may serve as a more stable salt template than NaCl.

The results observed here align with the findings of Xia et al. (2006) [43] who reported that the occupied mesopores exhibit reduced surface area, pore volume, and pore size compared to their open counterparts. Furthermore, the results are more consistent with the behavior of MBG rather than normal bioactive glass (BG), as demonstrated by Lei et al. [44].

**Figure 7:**
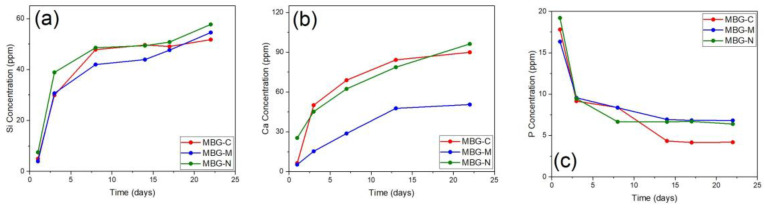
ICP-OES results showing the release of (**a**) silicon, (**b**) calcium, and (**c**) phosphorus ions from MBG-C, MBG-M, and MBG-N over a period of 22 days. The ion release shows the biomineralization process of MBG through the release of ions and the formation of calcium phosphate groups on the surface of bioactive glass.

The release of ions from MBGs is important to show the biomineralization process. This process involves the formation of the HA layer on the surface of the MBG. This layer is essential for bonding with bone tissue and promoting bone regeneration. ICP was used to study the leaching of different ions from the surface of MBG, including calcium (Ca^2+^), phosphate (PO_4_^3−^), and silicon (Si^4+^), which were released into SBF [45]. Figure 7 shows the ion release of silicon, calcium, and phosphorus ions from the different samples of MBG over 22 days. Figure 7a shows that, initially, silicon is rapidly released from all different MBGs as they are placed in SBF. It is also observed that MBG-N has the highest initial release, followed by the other two, as both show similar releases at that point. After the initial rapid release, the silicon concentrations continue to increase but more gradually, whereby at the end of the study MBG-N still shows the highest release, followed closely by MBG-M and MBG-C. Figure 7b shows the release of calcium ions, which increases over time for all MBG but especially for MBG-C, which shows the highest calcium release overall. MBG-N shows a more moderate steady release, and MBG-M shows the slowest release out of the three over the testing period. Figure 7c shows that all compositions exhibit an initial spike in phosphorus (P) concentration, followed by a sharp decrease as phosphorus is consumed to form the calcium phosphate layer (HA) on the surface of the MBGs [45]. This is consistent with the biomineralization process, where phosphorus ions from the solution react with calcium ions released from the glass to precipitate HA. The release of calcium (Ca^2+^), which is a network modifier ion, shows a rapid initial release, reaching a maximum concentration after only 5 days. This trend is consistent across different MBG compositions, which shows the role of calcium in promoting bioactivity. In contrast, the release of phosphorus is governed by its lower concentration in the glass network. As a result, phosphorus reaches its maximum concentration early in the release profile and is then consumed from the solution to form the calcium phosphate layer. The release of silicon (Si^4+^) also plays a critical role in the formation of a silica gel layer, which serves as a precursor for HA nucleation. Together, these processes contribute to the bioactivity of MBGs and their ability to bond with bone tissue [45].

Table 2 presents the vancomycin loading efficiency of the different MBG formulations with and without calcination. The MBG-C sample had a 61% loading efficiency, while MBG-M and MBG-N had loading efficiencies of 88% and 26%, respectively. Notably, MBG-N showed the lowest drug loading efficiency among the three samples. The reduced drug loading of MBG-N can be attributed to its smaller pore size compared to the other two formulations. Pore size is a critical factor in drug loading, as it directly influences the available surface area and volume for drug adsorption and loading [46]. Smaller pores may restrict the entry and retention of drug molecules, thereby reducing the overall drug loading. The model drug used in this study, vancomycin, is often the first choice for treating biofilm infections in implant-related infections [47]. However, vancomycin has a large particle size, which may hinder its ability to enter the pores of the bioactive glass.

Interestingly, MBG-N displayed higher bioactivity than the other formulations. This observation suggests a potential trade-off between drug loading capacity and bioactivity. The enhanced bioactivity of MBG-N might lead to accelerated biodegradation during the drug loading process, which could explain the lower drug loading. This phenomenon highlights the complex interplay between material properties and drug loading [36]. To further explain the potential of MBG-N as a biomaterial for drug delivery, it would be valuable to investigate pre-loading vancomycin during the synthesis process. This approach could provide insights into its loading capacity and release kinetics under different conditions, potentially optimizing its performance as a drug delivery system [48].

As this research continues to explore the use of salt templates in bioactive glass production, it is important to consider the potential interactions between salts and metal precursors. These interactions may significantly influence various properties of the resulting bioactive glass, including bioactivity, drug loading capacity, release profiles, and the ability to form scaffolds for bone tissue regeneration [21]. Understanding these complex relationships is essential for developing effective and multifunctional biomaterials for orthopedic applications.

**Figure 8:**
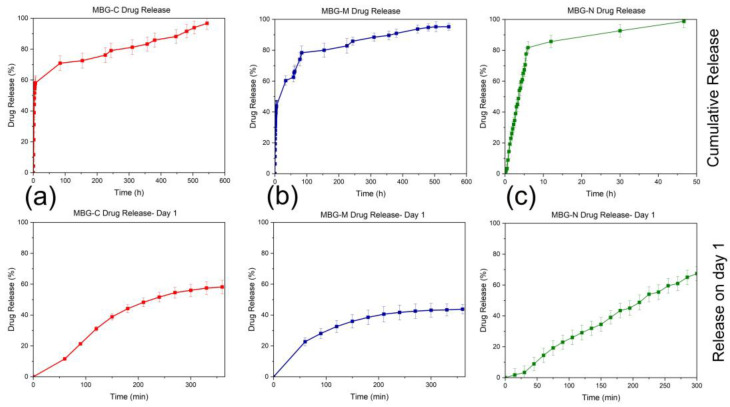
Vancomycin release from (**a**) MBG-C, (**b**) MBG-M, and (**c**) MBG-N in PBS. Each graph shows the release on day 1 as well as overall release which was measured until the release reached 100% or plateau. All the experiments were conducted in triplicate with an error rate of >5%.

Figure 8a shows the release profile of MBG-C, which initially has a burst release of the drug in the first six hours of the experiment, followed by a slower release over the rest of the testing period. This formulation offloaded around 60% of the loaded drug within the first 6 h and then released at a slower steady pace over the next 100 h, approaching ~95% release around 100 h. MBG-C was able to load around 61% of the drug by adsorption and was able to continue to release the drug as time progressed. That means that initially the drug adsorbed to the surface of the MBG was being released as a burst release, followed by the release of the drug entrapped deep within the pores of the MBG, which was released more slowly.

Figure 8b shows the release profile of MBG-M, which also indicates an initial burst release followed by a more sustained release over the testing period. In the first 6 h of the experiment, around 40% of the drug was released, followed by a gradual release of the remaining drug over the remainder of the experiment, reaching around 95% release around 600 h. As this formulation had the highest loading out of the three, it is predicted that the drug will keep releasing as the material biodegrades.

Figure 8c shows the release of MBG-N; this formulation had the lowest loading out of all of them and released all loaded drugs within 50 h of the start of the experiments. As with all the other formulations, there was a burst release initially followed by a slower release that lasted only a couple of days where it achieved 100% release. This formulation had the lowest loading rate, which means there was less drug initially, and it was probably adsorbed to the surface of the MBG, releasing as it was immersed in PBS. About 80% of the drug was released within the first 10 h, followed by a plateau that led to a slight increase in release over the next 40 h.

**Figure 9:**
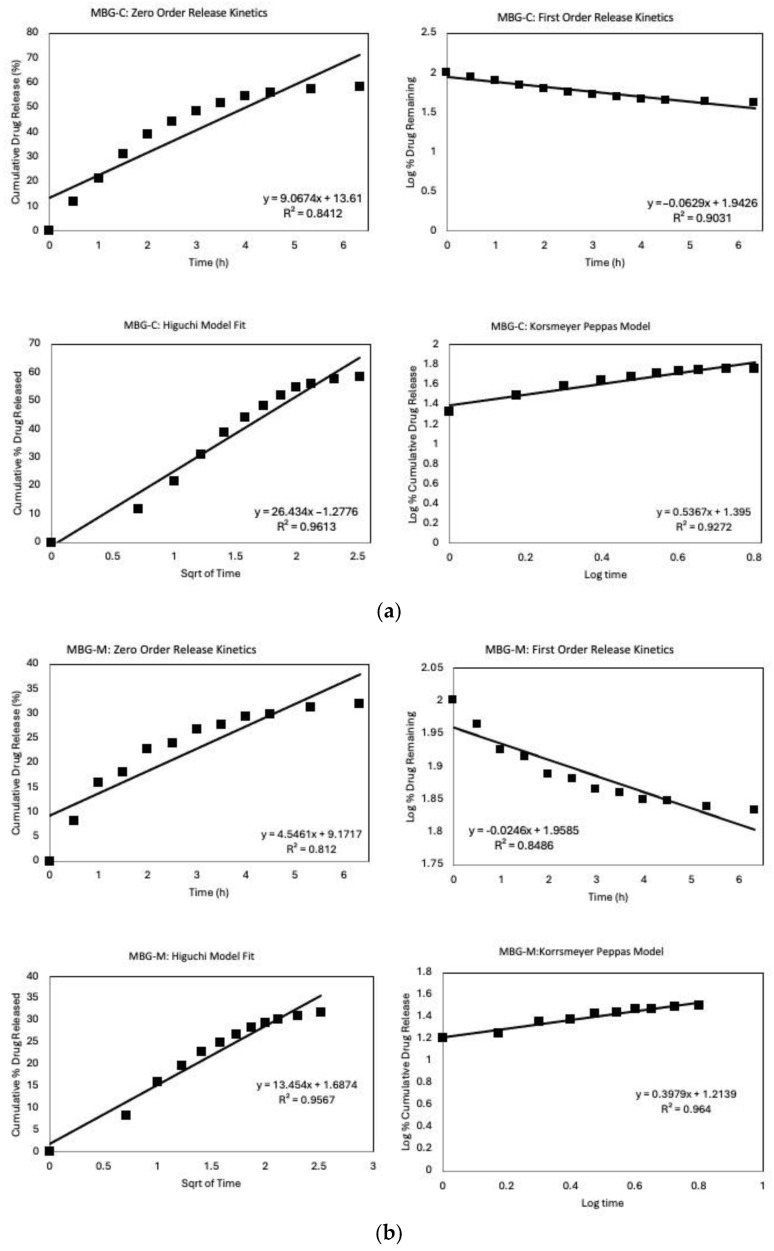

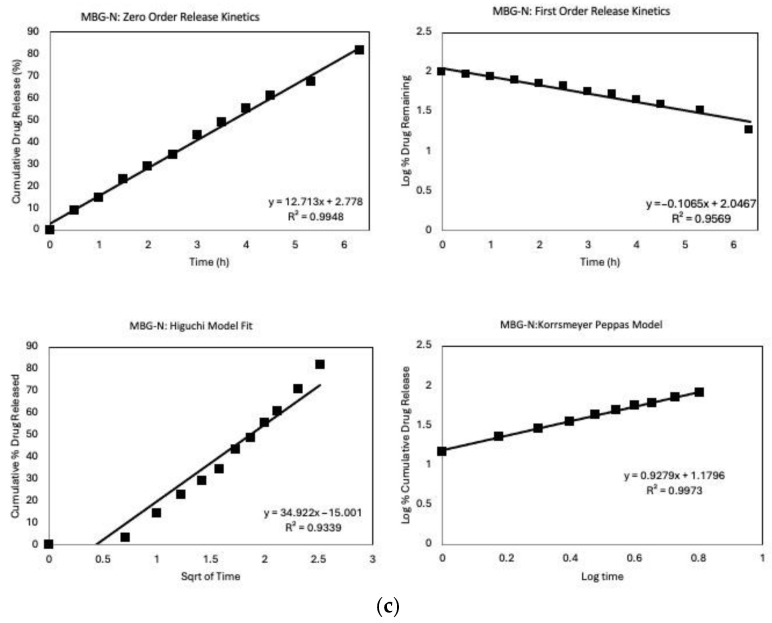
Release Kinetics graphs of the different MBGs that includes fitting the first 6 h into different models; zero-order, first-order, Higuchi, and Korsmeyer–Peppas models. (**a**) MBG-C model fit, (**b**) MBG-M model fit, and (**c**) MBG-N model fit.

In Figure 9, the drug release data were fitted using model-dependent methods based on the mathematical functions described earlier. Each graph represents the first six hours of release, fitted through zero-order, first-order, Higuchi, and Korsmeyer–Peppas models to determine which best describes the release of vancomycin from different MBG formulations. Figure 9a shows the model fitting for MBG-C, where the Higuchi model has the highest coefficient, R^2^, among the four models, indicating it best describes drug release from MBG-C. The fit of the Higuchi model for the MBG-C drug release data suggests a diffusion-controlled release mechanism, implying that the drug is dispersed throughout the material’s porous structure [30,36,49].

Originally developed for planar systems, the Higuchi model has been widely applied to various drug delivery systems, including porous matrices [50]. Its applicability to MBG systems shows the importance of diffusion in controlling drug release from these materials. This aligns with the fundamental characteristics of mesoporous materials, where the interconnected pore network facilitates drug diffusion [51].

Figure 9b shows that MBG-M has also been fitted through different drug release models, with the Korsmeyer–Peppas model providing the best fit. The release exponent, n, was found to be 0.397, which means that the material follows a Fickian diffusion of release. The fit indicates that drug release is governed by a standard diffusion process which is confirmed by the high R^2^ value fir of the Higuchi model as well, which again suggests a diffusion-controlled release mechanism [49]. Notably, MBG-M demonstrates a more sustained release profile compared to the other MBGs, making this formulation potentially ideal for long-term drug delivery applications [30]. Also, this formulation exhibited the highest drug loading capacity among the three compositions studied. This characteristic, combined with its sustained release profile, suggests that MBG-M could be especially useful for the delivery of antibiotics to treat persistent bacterial infections, such as those associated with biofilms [52]. Biofilms are known to be resistant to antibiotic treatment and often require prolonged exposure to therapeutic agents for complete eradication [53].

Figure 9c illustrates the model fit for MBG-N, where most models demonstrate a high R^2^. The high fit across the different models may be caused by the high initial release followed by the release of all the loaded antibiotics within the first few days of the study [54]. The zero-order model shows an excellent fit, suggesting that drug release is concentration-independent, which is unusual for porous systems and may indicate a complex release mechanism [55].

Diffusion still plays a crucial role in the release of vancomycin from MBG-N, as evidenced by the good fit of the Higuchi model [49]. However, the Korsmeyer–Peppas model provides the best fit among all models, indicating that the release mechanism is more complex than simple Fickian diffusion [56]. The excellent fit of the Korsmeyer–Peppas model suggests that the release follows anomalous transport, which involves a combination of Fickian diffusion and non-Fickian diffusion governed by the degradation of MBG [57]. The release exponent, n, is found to be 0.927 which means that it follows Super Case II transport confirming that the drug release is governed by MBG erosion and degradation.

The excellent fit of the Korsmeyer–Peppas model, coupled with the rapid and complete drug release, implies that the release mechanism may involve the degradation of the MBG simultaneously with drug diffusion [58]. This simultaneous process of drug release and MBG degradation is particularly relevant for bioactive materials designed for tissue engineering applications, where the gradual degradation of the MBG can promote tissue regeneration while delivering therapeutic agents [30].

When comparing the different MBGs, it is observed that each of the glasses displays a different release mechanism that can be used to achieve different treatment goals; some of the formulations will release all loaded antibiotics within the first couple of days while others will last for around a month. However, drug loading and release are highly influenced by the surface properties of MBG. Hence, the enhanced drug loading capacity of MBG-M may be attributed to the presence of some Mg(OH)_2_, which can alter the surface properties and pore structure of the mesoporous bioactive glass [59]. This modification likely influences the drug–matrix interactions, potentially leading to higher drug retention and more controlled release [22].

**Figure 10:**
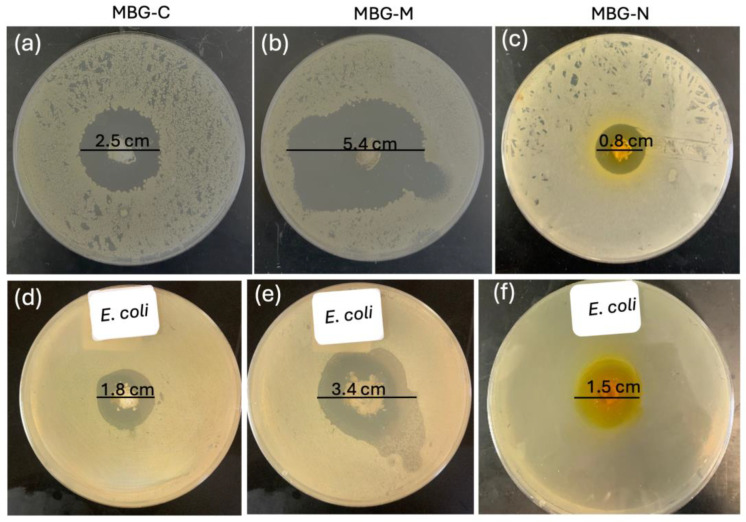
Disc diffusion test that shows the zone of inhibition of *S. aureus* using vancomycin-loaded MBG. (**a**) shows the ZOI of 2.5 cm clearance for MBG-C, (**b**) shows a ZOI of 5.4 cm for MBG-M, and (**c**) shows a ZOI of 0.8 for MBG-N. The zones of inhibition for *E. coli* are shown in (**d**) for MBG-C with a ZOI of 1.8 cm, (**e**) for MBG-M with a ZOI of 3.4 cm, and (**f**) for MBG-N with a ZOI of 1.5 cm. The zone of inhibitions were recorded after 24 h.

Figure 10 shows the antibacterial efficacy of vancomycin-loaded MBGs evaluated using the well agar diffusion method against *S. aureus* which is a Gram-positive bacterium commonly found on the skin and prosthetic joint infections [16]. The MBGs were loaded into wells on agar plates, and the zones of inhibition (ZOI) were measured after 24 h of incubation at 37 °C. Figure 10a shows a 2.5 cm ZOI for MBG-C which means that the MBG was able to successfully clear bacteria by releasing vancomycin. Figure 10b shows the ZOI for MBG-M which was measured to be 5.4 cm meaning that MBG-M is very effective against *S. aureus*. Figure 10c shows the ZOI for MBG-N which is the smallest out of the three with only 0.8 cm clearing meaning that MBG-N is also effective against the bacteria.

Figure 10 also tests antibacterial activity against Gram-negative *E. coli*. In Figure 10d, MBG-C shows a ZOI of 1.8 cm against *E. coli*. Figure 10e demonstrates MBG-M’s stronger effect, with a larger ZOI of 3.4 cm, proving it works equally well against both Gram-negative and Gram-positive bacteria. Finally, Figure 10f reveals MBG-N has a ZOI of 1.5 cm against *E. coli*, showing it is more effective against Gram-negative bacteria than Gram-positive ones.

**Figure 11:**
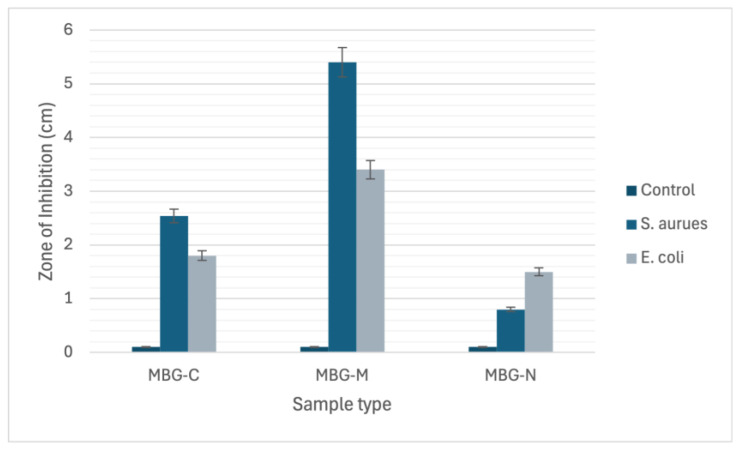
The chart graph depicts the zone of inhibition (ZOI) measurements for MBG-C, MBG-M, and MBG-N against both *S. aureus* and *E. coli* bacterial strains.

The differences among the three MBGs can be attributed to differences in drug loading, pore size, surface area, and biodegradability. MBG-M had the highest drug loading among the three and had the largest ZOI which was more significant than the other two samples as depicted in Figure 11. MBG-C had the second highest drug loading and the second largest ZOI, and finally MBG-N had the lowest drug loading, but it was still able to inhibit bacterial growth.

## 3. Materials and Methods

For the synthesis of mesoporous bioactive glass, tetra orthosilicate (TEOS) was used as the silicon source, tetraethyl phosphate (TEP) as the phosphorous source, and calcium nitrate tetrahydrate (CaN) as the calcium source. The pores were created using the salts magnesium hydroxide Mg(OH)_2_ and NaCl (Sigma-Millipore, Oakville, ON, Canada), and all synthesis occurred in Millipore ultrapure water. Tris (hydroxymethyl) aminomethane (TRIS) was used to maintain the solution at a pH of 8 and hydrochloric acid was used to wash the salt out of the pores. Phosphate-buffered saline (PBS) tablets (Sigma-Millipore, Oakville, ON, Canada) dissolved in deionized water were used for the drug release studies. Vancomycin hydrochloride (sterile for IV injection, USP) used in the experiment was generously gifted by Mount Sinai Hospital, Toronto, Canada. The materials were all purchased from Sigma-Millipore, Canada and were used without further purification or modifications.

### 3.1. Experimental Procedure for Synthesizing MBG

Three different MBG samples were synthesized using Mg(OH)_2_ salt as a template; the first sample was treated thermally through calcination at 600 °C (named MBG-C), the second sample was also synthesized with using Mg(OH)_2_ salt but was used without calcination (named MBG-M), and finally a reference sample was made with ethanol-insoluble NaCl which was named MBG-N and was used without calcination.

The synthesis of MBG was conducted in a 60 mL solution of TRIS buffer and D.D.I water with a pH of 8. Mg(OH)_2_ and NaCl salts of 1 g were added to the solution and dispersed through continuous mixing for at least one hour to ensure complete dispersion of the salt particles. Subsequently, TEOS, TEP, and CaN were sequentially added to the solution, with a one-hour interval between each addition to facilitate the hydrolyzation and condensation of the materials. The mixture was then stirred at 250 rpm at room temperature overnight to allow for the formation of the MBG structure.

**Figure 12:**
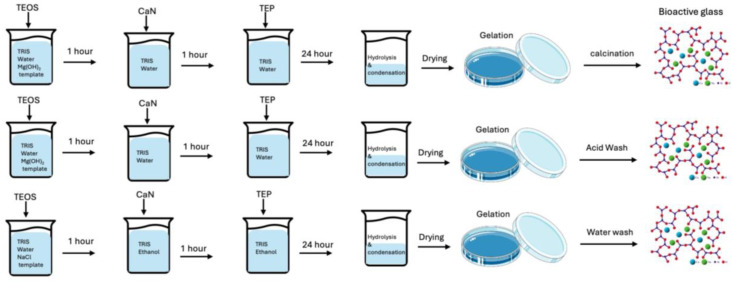
Schematic illustration of bioactive glass synthesis using salt templating. The diagram compares the synthesis processes employing Mg(OH)_2_ and NaCl as templates, highlighting the distinct template removal methods (calcination, acid washing, or water washing).

The salt template was removed by calcination at 600 °C for 4 h, which is higher than the melting temperature of Mg(OH)_2_ [60]. Calcination is an important step in the synthesis of bioactive glass as it leads to the removal of unwanted or residual remnants of the hydrolysis and condensation reactions [12]. The salt template was also removed by washing the formed MBG with mild hydrochloric acid dropwise with 1M concentration. This addition of HCl is followed by drying and washing of the powders to eliminate any residual HCl as shown in Figure 12. Synthesized MBG samples were subjected to various analyses, including X-ray diffraction, Fourier-transform infrared spectroscopy (FTIR), scanning electron microscopy (SEM), and BET analysis, to characterize its composition and structure.

### 3.2. Characterization

#### 3.2.1. X-Ray Diffraction

X-ray diffraction patterns were collected for all MBG samples in order to characterize their amorphous structure and to investigate if the hard templating was successful. A MiniFlex 600 diffractometer (Rigaku Corporation, Tokyo, Japan) was used to collect X-ray dispersive patterns under Cu Kα radiation (40 kV, 15 mA) to determine crystallinity. The instrument was set to take measurements between 10 and 60 °C at an increment of 2 °C.

#### 3.2.2. Fourier-Transform Infrared Spectroscopy (FTIR)

The structure and bioactivity of different MBGs were investigated using a Cary 630 spectrometer (Agilent, Santa Clara, CA, USA) to assess molecular vibrations using the KBr pellet method from 4500 to 400 cm^−1^, before and after immersion in SBF. The MBG samples were immersed in SBF to evaluate the growth of HA nanocrystals on the material surface as a measure of bioactivity. Each glass sample was filtered and dried at predetermined times and then analyzed using FTIR following protocols established in previous studies [1,61].

#### 3.2.3. Inductively Coupled Plasma Optical Emission Spectrometry (ICP-OES)

An Agilent 5900 system (Agilent, Santa Clara, CA, USA) was used to analyze ion concentrations released into the SBF at various time points over 21 days to study degradation of the MBG samples. ICP-OES was used to measure the degradation and bioactivity of the highly porous bioglass synthesized in this study. It consists of analyzing the concentrations of calcium, phosphorus, and silicon following the placement of bioglass in SBF for different periods. In this study, 2.5 mL of the sample was removed and added to nitric acid to eliminate potential interference and contamination. The samples were collected on days 1, 3, 5, 10, 15, and 21 to show the overall ion release trends. ICP-OES is used to determine the presence of elements in a sample which in the case of MBG will show the products of degradation over time through the release of silicon, calcium, and phosphorus [15].

#### 3.2.4. Microstructural Characterization

The surface area, pore volume, and pore size were measured for the MBG samples using a TriStar 3000 porosimeter (Micromeritics, Norcross, GA, USA). The Braunauer–Emmett–Teller (BET) model was used to find the surface area while the Barrett, Joyner, and Halenda (BJH) was used to find pore volume and size and show the desorption isotherm. A sample of 0.1 g was used for analysis, with each sample degassed at 200 °C for 12 h to clear up the pores and allow for accurate measurements.

#### 3.2.5. Scanning Electron Microscopy (SEM) and Energy Dispersive X-Ray Spectroscopy (EDX)

SEM JSM-6380LV (JEOL, Tokyo, Japan) was used to characterize the morphology and structure of the samples after SBF exposure. The bioglass samples were immersed in SBF at a ratio of 1.5 mg/mL and left in a water bath in a shaker at 37 °C for several days. The samples were then filtered and air-dried to investigate the development of HA on the surface. The same instrument was used to perform EDX analysis to investigate the elemental composition of the synthesized glass after immersion in SBF for several days.

### 3.3. In Vitro Bioactivity

A simulated body fluid (SBF) solution was prepared by dissolving reagent-grade NaCl, KCl, NaHCO_3_, MgCl_2_·6H2O, CaCl_2_, and KH_2_PO_4_·3H_2_O in distilled water. The solution was buffered at pH 7.4, using Tris (hydroxymethyl) aminomethane (TRIS) and 6N HCl at 37 °C, according to the Kokubo protocol [34]. The biomimetic SBF solution had an ionic composition similar to human blood plasma. This enables the in vitro evaluation of bioactive glass dissolution and apatite formation under physiologically relevant conditions [15,34]. To assess dissolution, 15 mg of MBG powder was suspended in 10 mL of SBF at a ratio of 1.5 mg/mL and kept at 37 °C with constant stirring in a water bath shaker for 30 days. The tubes were then centrifuged and filtered to recover MBG, which was fully dried prior to analysis by FTIR spectroscopy. The supernatant was analyzed using ICP-OES, as described below.

### 3.4. Drug Loading and In Vitro Release Profile

Vancomycin was used as a model drug and loaded onto the carriers using a simple impregnation method [43,62]. Briefly, 200 mg of the MBG samples was added to 20 mL of antibiotic solutions prepared at 10 mg/mL in sterilized deionized water. The mixtures were stirred continuously for 24 h to facilitate drug adsorption onto MBG particles. The MBG samples were then filtered and the residual concentrations of vancomycin in the filtrate were measured using pre-calibrated UV-visible spectroscopy (Genesys 10S UV-Vis Spectrophotometer; Thermo Fisher Scientific, Madison, WI, USA) at wavelengths of 280 nm, to determine depletion [63,64,65].

A UV-Vis standard calibration curve for the vancomycin was established using stock solutions to determine the concentration of the remaining vancomycin after loading. According to the Beer–Lambert Law, the linear range of 20–600 μg/mL was obtained with a correlation coefficient of 0.99. The percentage of antibiotics loaded into the bioglass samples was calculated by comparing the initial and final concentrations using the following equation:

$$\mathrm{Loading}\;\mathrm{efficiency}\;\left(\%\right)=\frac{\mathrm{Ci}-\mathrm{Cf}}{\mathrm{Ci}}\times 100\%\;$$

where C_i_ and C_f_ are the initial and final concentrations, respectively.

Then, 50 mg glass samples were placed in 10 mL of phosphate buffer saline (PBS) in plastic tubes. The tubes were then placed in a shaker at 37 °C with continuous stirring for 600 h or until drug release reached 100% to mimic the physiological conditions. At 15 min intervals for the first day and then 1-day intervals, 2 mL of the release medium was collected for analysis using UV-visible spectroscopy to quantify the amount of drug released. To maintain the drug concentration gradient driving the release, the removed volume was replenished with an equal volume of fresh PBS solution.

To evaluate the vancomycin release mechanisms and kinetics from the MBG samples in vitro, cumulative release data were fitted to various mathematical models [27,66]. By comparing the coefficient of determination, R^2^, values to different models, the predominant release kinetics can be determined. The models applied in this study include:

Zero-order model: Assumes uniform drug release rate independent of concentration. It is calculated using the following equation:

$$C_{t}={\;C}_{0}+\mathrm{kt}$$

C_t_ is the amount of drug dissolved at time t.

C_0_ is the initial amount of drug in solution.

k describes the zero-order rate constant.

First—order model: Release rate dependent on concentration which is represented in a linear way. The following equation is used to calculate it:

$$\frac{\mathrm{dC}}{\mathrm{dt}}=-\mathrm{kC}$$

The above equation can be re-written as follows:

$$\log\;C\;={\log\;C}_{0}-\frac{\mathrm{Kt}}{2.303}$$

C_0_ is the initial drug concentration.

k describes the first-order rate constant.

Higuchi model: Describes drug release from matrix systems based on Fickian diffusion. It is calculated using the following equation:

$$f_{t}=Q=A\sqrt{D\left(2C-C_{s}\right)C_{s}t}$$

where Q refers to the amount of drug released in time t. A is the unit area, C is the drug initial concentration, and the drug solubility is represented by C_s_. D refers to the diffusivity of the drug [67].

The Higuchi model can be simplified to describe drug release from matric and polymeric systems as follows:

$$\frac{M_{t}}{M_{\infty }}=k\surd t$$

where M_t_/M_∞_ is the cumulative amount of drug released at time t, and k is the Higuchi constant.

Korsmeyer–Peppas: Empirical model combining diffusion and erosion effects. It is calculated using the following equation:

$$\frac{M_{t}}{M_{\infty }}={k\text{'}t}^{n}$$

where M_t_/M_∞_ is the cumulative amount of drug released at time t, k’ is the kinetic constant, and n is usually used to describe a specific diffusion mechanism. The exponent n is used to describe the diffusion mechanism of the system; where it represents Fickian diffusion if n = 0.5, non-Fickian transport if 0.45 < n = 0.89. If n = 0.89 then it is zero-order release (case II) transport. If n is higher than 0.89 then diffusion mechanism is Super Case II transport [67].

Fitting the vancomycin release profiles to these models enables identification of the predominant kinetics whether they be concentration-dependent, diffusion-based, or relaxational. Furthermore, the impact of composition and surfactant templating on the release mechanism can be explained by comparing model fitting between MBG formulations.

### 3.5. Antimicrobial Susceptibility Test

The antibacterial activity of vancomycin in MBG- C, MBG-M, and MBG-N was evaluated against *Staphylococcus aureus* (ATCC 25923) using the disc diffusion method as described in the British Society for Antimicrobial Chemotherapy (BSAC) Disc Diffusion Method for Antimicrobial Susceptibility Testing, Version 4 (2005) [68]. Hinton Mueller agar plates were used as the testing medium. *S. aureus* was prepared by suspending it in saline solution to achieve a suitable inoculum density. A 100 aliquot of bacterial inoculum was uniformly spread onto the surface of agar plates and allowed to dry for 5 min to ensure proper adhesion. After drying, 50 mg of each MBG sample was placed into wells on agar plates. The plates were then incubated at 37 °C for 24 h to facilitate bacterial growth and assess antibacterial interactions. Following incubation, the antibacterial efficacy was evaluated by measuring the zone of inhibitions (ZOI) around each MBG sample.

## 4. Conclusions

This study explored the impact of using inorganic salt as a template and calcination on the properties of MBGs. Salt templating successfully produced MBGs, as confirmed by XRD and FTIR patterns, which included the patterns of salt integrated in the structure before washing and its absence after washing it. Calcination showed enhanced bioactivity of MBGs, with MBG-C showing higher bioactivity and faster degradation compared to MBG-M and MBG-N. MBG-M exhibited the highest surface area and pore volume after washing, along with maximum drug loading and the slowest release rate, making it an excellent candidate for prolonged antibiotic delivery applications. In contrast, MBG-C showed a high surface area but lower drug loading capacity and faster release, making it suitable for applications requiring higher drug concentrations and bone regeneration. Although MBG synthesis using NaCl was successful, MBG-N displayed a relatively low surface area, drug loading efficiency, and rapid release. These findings highlight that the properties of bioactive glasses are highly dependent on the synthesis methods, and any changes in the procedure will certainly influence the material’s characteristics.

The results demonstrate that synthesizing MBGs without calcination is possible, and the use of easily removable templates is successful. MBG-M exhibited remarkable properties, showing great potential for long-term drug delivery in bone regeneration applications. It is important to understand the influence of calcination on MBG structure, as this study highlights that thermal treatment significantly impacts properties such as bioactivity, biodegradation, and surface area. The lower drug loading of MBG-C may suggest a collapse in the pore structure during calcination. Synthesizing MBGs without calcination opens new possibilities for applications requiring extended drug release and slower biodegradation, allowing bones to regenerate around defects effectively.

## Figures and Tables

**Table 1:** Surface area, pore volume, and pore size of the synthesized MBG before and after washing and calcination.

MBG	Surface Area (m^2^/g)	Pore Volume (cm^3^/g)	Pore Size (nm)
MBG-C	201.41	0.33	6.56
MBG-M before washing	74.37	0.14	3.75
MBG-M after washing	426.91	0.36	7.41
MBG-N before washing	46.59	0.14	5.75
MBG-N after washing	174.24	0.26	5.96

**Table 2:** Drug loading of different MBG calcination and washing. All experiments were conducted in triplicate, and the results are presented with the standard error.

MBG	Loading Efficiency (%)
MBG-C	61 ± 5.12
MBG-M	88 ± 1.76
MBG-N	26 ± 3.12

## Data Availability

Available upon request.

## Abbreviations

The following abbreviations are used in this manuscript:

BET	Braunauer–Emmett–Teller
BJH	Barrett, Joyner, and Halenda
CMC	Critical micelle concentrations
EDX	Energy Dispersive X-ray
FTIR	Fourier-transform infrared spectroscopy
HA	hydroxyapatite
HCl	Hydrochloric acid
ICP-OES	Induced Coupled Optical Emission Spectroscopy Plasma
MBG	Mesoporous bioactive glass
MBG-C	Calcined mesoporous bioactive glass
MBG-N	Mesoporous bioactive glass-NaCl
MBG-M	Mesoporous bioactive glass-Mg(OH)2
NBO	Non-bridging Oxygens
SBF	Simulated body fluid
SDA	Surface-directing agents
SEM	scanning electron microscopy
XRD	X-Ray Diffraction
ZOI	Zone of inhibition
